# The Impact of Storage Conditions on Peanut Seed Quality, Growth, and Yield

**DOI:** 10.3390/plants14192944

**Published:** 2025-09-23

**Authors:** Puxiang Shi, Hongxi Sun, Yibo Wang, Ning Han, Liang Ren, Jinhao Lv, Qing Guo, Kang He, Haixin Wang, Guoqing Yu

**Affiliations:** 1 Liaoning Research Institute of Sandy Land Control and Utilization, Fuxin 123000, China; spx_104@126.com (P.S.);; 2 Shandong Peanut Research Institute, Qingdao 266100, China; 3 State Key Laboratory of Nutrient Use and Management, Shandong Academy of Agricultural Sciences, Jinan 250100, China

**Keywords:** peanut, seed quality, storage conditions, cold stress, yield

## Abstract

As one of the major oil crops worldwide, peanuts play a crucial role in ensuring the stability of global oil production and quality. Seed quality, a direct determinant of yield, is influenced by various factors, among which storage temperature and moisture content are critical. However, the mechanisms by which storage conditions affect peanut seedling development and final yield remain unclear. To address this, we conducted field plot experiments using different storage temperature regimes (0 °C, −10 °C, −20 °C, −40 °C) and seed moisture contents (5%, 10%, 15%) to evaluate their effects on seed quality, subsequent growth, and yield. The results showed that, at the same storage temperature, seed vigor declined with increasing seed moisture content. Conversely, at the same seed moisture content, seed vigor decreased with lower storage temperatures. Overall, the highest germination rate (99.21%) and emergence rate (96.79%) were observed under the 0 °C/5% treatment. Nutrient composition analysis revealed that, at a constant storage temperature, protein content was negatively correlated with seed moisture content, whereas linoleic acid content was positively correlated. After sowing, antioxidant enzyme activities in leaves were monitored throughout seedling development. Enzyme activities initially increased and then declined as plants matured. At the early seedling stage, the highest activities of superoxide dismutase (SOD) and peroxidase (POD) were detected under the 0 °C/5% treatment. In contrast, malondialdehyde (MDA) content increased significantly with decreasing storage temperature and increasing seed moisture content. From a yield perspective, these factors collectively influenced yield components under different treatments, with the maximum yield (6187.5 kg/ha) obtained under the 0 °C/5% treatment. In summary, the increase in nutrient content and peroxidase activity during the seedling stage of peanut seeds treated with 0 °C/15% water content improved seed quality and vitality, making seed preservation more suitable under these conditions. On the other hand, we conducted transcriptome sequencing on peanut varieties with different cold tolerance levels and identified a cold tolerance gene AhCOLD1, which was preliminarily validated to be involved in cold stress response. In summary, we have determined the optimal storage method for local peanut seeds and identified a cold resistant gene, providing effective technical support for stabilizing local peanut production.

## 1. Introduction

As a major global oil crop, the stable and normal growth of peanuts plays an important role in maintaining global oil supply [1,2]. As the first step in peanut cultivation, the quality of seeds has a decisive impact on the stable inheritance of excellent peanut traits and the guarantee of yield [3]. In agricultural production, seeds harvested this year are usually stored and used for production the following year. Therefore, maintaining good seed quality is a key indicator affecting the continuous and stable production of peanuts [4].

Seed vitality is usually influenced by various factors, including species, environmental conditions, planting methods, seed maturity, and storage methods, among others [5]. The storage temperature and seed moisture content are key indicators that affect seed vitality [6]. In general, the lifespan of seeds increases with the decrease in storage temperature, but excessively low temperatures can cause freezing damage to seeds, which in turn affects their vitality [7]. Currently, common seed storage temperatures range from 0 °C to −40 °C, which are mainly influenced by plant type, growth cycle, and planting environment. For example, Mbofung et al. (2013) [8] reported that soybean seeds had better seed vitality when stored at 10 °C; Arundhati et al. (2012) [9] found that the seeds of gum karaya (*Sterculia urensRoxb.*) were best stored below 0–4 °C; Guo et al. (2025) [10] found that storing Elaeagnus Mollis at −20 °C is more beneficial for extending its seed lifespan and maintaining its vitality.

The moisture content of seeds also affects their vitality, and excessively high or low moisture content can directly lead to premature germination or loss of germination ability [11]. For example, when the moisture content of onion seeds reaches 15%, their germination rate drops sharply, significantly lower than the control level [12]. It is interesting that different plant types have different requirements for seed moisture content. For example, lettuce seeds maintain good vitality even at a moisture content of 15%, and further research has shown that the fat content in lettuce seeds is directly related to seed vitality. On the contrary, barley seeds can still maintain vitality at a moisture content of up to 28%, significantly higher than the moisture content of lettuce seeds [13,14].

The seeds of oil crops themselves contain a large amount of fat, and compared to other seeds, their quality is more easily affected by storage conditions. On the one hand, improper storage can lead to the production of toxic substances such as aflatoxins in the seeds, directly affecting the safety of edible oil [15]. On the other hand, unreasonable storage conditions can lead to a decrease in seed vitality, which in turn leads to a decrease in yield in the second year and seriously increases agricultural planting costs. For example, some scholars have reported [16] that drying soybeans on farms or in warehouses without air conditioning can lead to repeated drying and wetting of seeds, affecting seed quality. Therefore, they used a fan to adjust the moisture content of the seeds to ensure their quality. However, due to the diverse types of oil crops and the differences in planting locations, there are currently no fully applicable seed storage methods, and adaptability tests are conducted based on specific local planting varieties and ecological environments. However, overall, temperature and moisture content remain one of the most important influencing factors [17].

Buitink et al. (2000) [18] and Jaganathan et al. (2024) [19] found that different storage temperatures have a relatively suitable moisture content to ensure the maintenance of basic biochemical reactions of seeds, etc. Peanuts, as a widely adaptable oil crop, have a wide distribution of cultivable areas and different climatic characteristics in different regions. In planting bases with cold storage and professional storage, peanut seeds are dried after harvesting and then stored in refrigerators and cold storage facilities. In agricultural growers, they are usually dried and stored directly in warehouses, and the specific environmental temperature and humidity are mainly affected by weather conditions. For example, in the northeastern region of China, rapid cooling weather such as frost is prone to occur in spring [20]. Therefore, studying the mechanisms of the effects of different temperatures and moisture contents on seed germination and peanut growth is an effective direction for finding suitable local seed storage conditions and planting plans.

At present, peanuts mainly resist cold stress through two ways: molecular defense and physiological defense, the most typical of which include increased peroxidase activity and upregulation of key cold resistant genes. Among them, SOD, POD, and catalase (CAT), as three typical peroxidase systems, have been widely reported [21]. In terms of molecular mechanisms, since the cloning of the cold tolerance gene *COLD1* in rice in 2015 [22], its role as a regulator of the G protein signaling pathway to enhance rice cold tolerance has gradually become clear [23,24]. Subsequently, the *COOL1* gene was also discovered in maize [25], and natural variations in its promoter region regulated maize’s response to low temperatures by affecting the binding of the key transcription factor HY5 in light signaling, revealing a new mechanism for maize adaptation to high latitude and low temperature environments [26]. *COLD1* and its homologous genes play an important role in the perception and transmission of low-temperature signals in different plants, mainly by interacting with G proteins to activate calcium ion channels and trigger downstream cold resistance defense responses [24]. However, in the field of peanut research, although the *AhCOLD1a* and *AhCOLD1b* genes have been cloned in this study, their functional verification and molecular mechanism analysis are still in their early stages compared to crops such as rice and corn.

In order to find better storage conditions, we studied the two main influencing factors of storage temperature and moisture content as variables to explore the optimal storage conditions for peanut seeds. We designed different moisture contents (0.5%, 10%, 15%) and storage temperatures (0 °C, −10 °C, −20 °C, −40 °C) to study their effects on peanut seed vitality and final yield. The purpose of this experiment is to provide technical support for the development of more suitable seed preservation plans in the local area by studying physiological and molecular changes.

## 2. Results

### 2.1. The Effects of Storage Temperature and Moisture Content on Seed Vitality and Emergence

**Figure 1:**
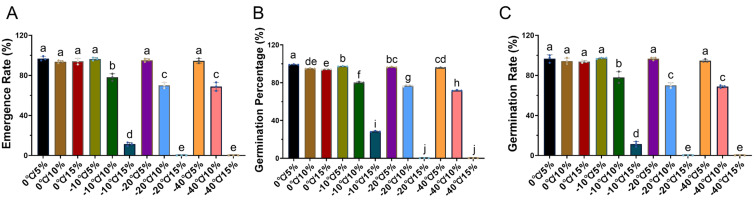
Effects of low-temperature storage under different seed moisture contents on peanut seed germination and vigor. Peanut seeds with three different moisture contents (5%, 10%, and 15%) were stored at four low temperatures (0 °C, −10 °C, −20 °C, and −40 °C) for 2 months, resulting in 12 treatments (e.g., 0/5 indicates storage at 0 °C with 5% seed moisture content). After storage, seed germination was evaluated under controlled incubator and field conditions. The measured indices were: (**A**) emergence rate (ER%), (**B**) germination potential (GP%), and (**C**) germination rate (GR%). Data are presented as mean ± standard deviation (SD) of three biological replicates. Significant differences among treatments were determined using one-way ANOVA followed by Tukey’s HSD test (*p* < 0.05). Different lowercase letters above the bars indicate significant differences among treatments.

The changes in moisture content and temperature significantly affect the vitality and quality of seeds. Firstly, from the perspective of temperature, the germination of all treated seeds reached over 93% at 0 °C, with the maximum of 96.79% obtained at 5% moisture content, while other moisture treatments showed a slight decrease (Figure 1). Germination significantly decreased with increasing moisture content at −10 °C, reaching a minimum of 11.3% at 15%. The changes at −20 °C and −40 °C were basically the same, both declining sharply as moisture content increased. In particular, seeds failed to germinate (0%) at 15% moisture under these two temperatures. On the other hand, in terms of moisture content, seeds under different temperature treatments showed germination above 94.6% at 5% moisture. As moisture content increased, germination decreased significantly. Interestingly, the effect of moisture content at 0 °C was relatively small, decreasing by only about 2%. Overall, germination was highest under the 0 °C/5% treatment. Similarly, the trends in germination potential and emergence rate followed a similar trend to germination, both peaking at 0 °C/5% and failing to occur at −20 °C/15% and −40 °C/15%.

### 2.2. Effects of Storage Temperature and Moisture Content on Seed Nutrients

The germination and stress resistance of seeds require their own provision of nutrients, so we further analyzed the nutrient content of seeds under different storage conditions (Table 1). The results showed that the protein content gradually decreased with the increase in water content at 0 °C and −10 °C, but the decrease was smaller at 0 °C (0.27%), while the decrease was more significant at −10 °C (0.63%). On the other hand, the protein content first increased and then decreased with the increase in water content at −20 °C and −40 °C, with significant fluctuations (0.37–1.22%). At the same time, the fat content decreased with increasing water content under different temperature treatments, and the decrease in fat became more pronounced with decreasing temperature. Overall, the minimum value was found at −40 °C/15%. The trend of changes in oleic acid, linoleic acid content, and fat content followed a similar decreasing trend, both decreased with increasing water content. It is interesting that as the storage temperature decreases and the moisture content increases, the ratio of linoleic acid to oleic acid an increase of 15.2%, and at a moisture content of 15%, the ratio exceeds 1, especially in the −40 °C/15% treatment where the ratio reaches 1.2.

### 2.3. Effects of Storage Temperature and Moisture Content on Peanut Antioxidant Enzyme Activity

**Figure 2:**
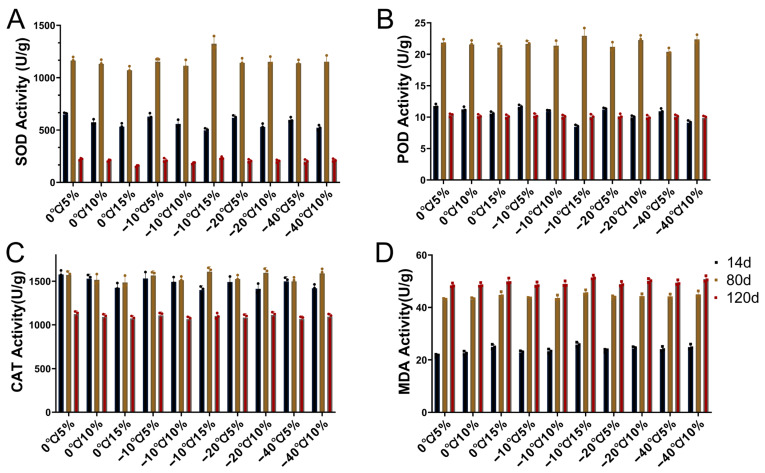
Antioxidant enzyme activities (**A**–**C**) and malondialdehyde (MDA) content (**D**) in samples stored under different temperature and relative humidity (RH) conditions at three time points. (**A**) Superoxide dismutase (SOD) activity, (**B**) Catalase (CAT) activity, (**C**) Peroxidase (POD) activity, and (**D**) Malondialdehyde (MDA) content were measured after 14, 80, and 120 days of storage under various combinations of temperature (0 °C, −10 °C, −20 °C, −40 °C) and RH (5%, 10%, 15%). Data are presented as means ± standard deviations (n = 3).

To investigate the effects of different storage conditions on seed germination and growth, we measured the antioxidant enzyme activity of peanut leaves at the early, middle, and late stages (Figure 2). The results showed that in the early stage (14 days), the activity of SOD in leaves increased with increasing water content at the same temperature, reaching a maximum value in the 0 °C/5% treatment. In the medium term (60 days), SOD activity exceeded 1000 U under all treatments, with the highest value in the −10 °C/15% treatment. In the late stage (80 days), SOD activity decreased in all treatments to below 250 U, which was lower than that observed in the early stage. Across different treatments, SOD declined with increasing water content at 0 °C and reaching its minimum at 15% water content. At −10 °C, SOD activity first decreased and then increased, whereas it changed little at −20 °C and −40 °C.

Similarly, POD content in the early stage decreased with increasing water content at the same temperature, with a maximum at 0 °C/5% and a minimum at −10 °C/15%. In the middle stage, POD content increased, generally rising with water content except for the 0 °C treatment, with a maximum at −10 °C/15%. In the late stage, POD content returned to the initial level and decreased with increasing water content at the same temperature. CAT activity followed a trend similar to that of POD, reaching maximum values in the middle stage before decreasing in the late stage. Notably, changes in CAT content were minor in the early and middle stages (1.5–12.1%). Moreover, increasing moisture content reduced the increase in POD. Overall, as storage temperature decreased and moisture content increased, antioxidant enzyme activity in peanut leaves showed a declining trend.

We also measured the content of malondialdehyde (MDA) as an indicator of the degree of cell membrane damage. As shown in Figure 2D, MDA content in peanut leaves increased with decreasing storage temperature or increasing moisture content. After 14 days of storage, the lowest MDA content was observed under the 0 °C/5% treatment, whereas the highest was observed under the −10 °C/15% treatment, representing a 19.6% increase relative to the 0 °C/5% treatment. In addition, MDA content was lowest in leaves at the early seedling stage and increased progressively during seedling development.

### 2.4. Positive Effects of Appropriate Storage Temperature and Moisture Content on the Yield of the Next Generation of Plants from Seeds

**Figure 3:**
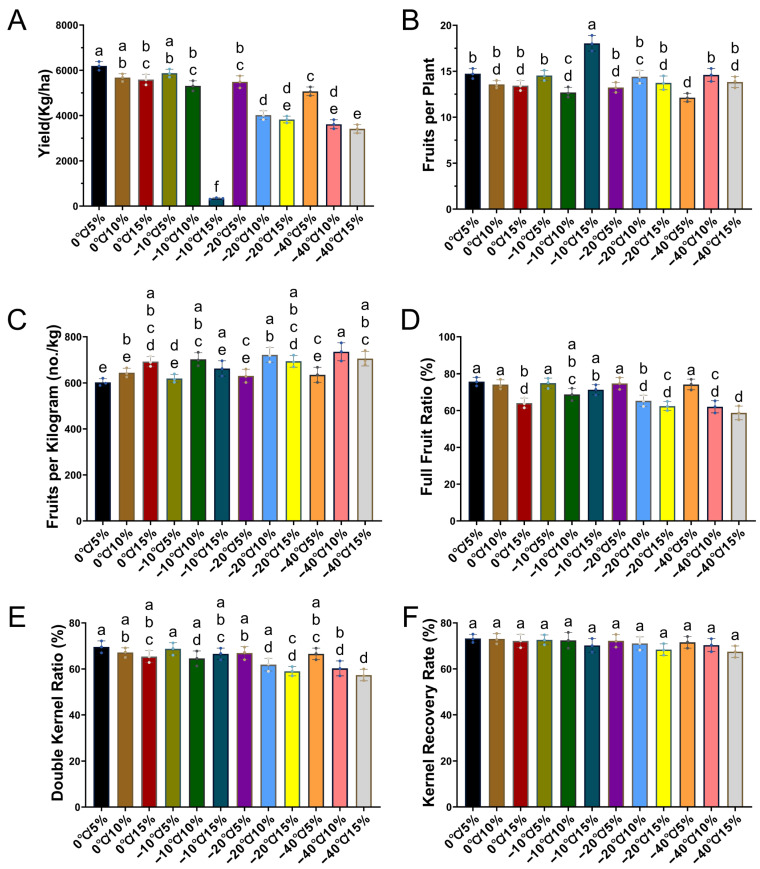
Phenotypic performance of peanut under different temperature and moisture treatments. (**A**) Yield: measured as the total seed weight per plot converted to kg per ha. (**B**) Number of fruits per plant: counted from randomly selected representative plants. (**C**) Fruits per kilogram: calculated as the number of fruits per unit weight. (**D**) Full fruit ratio (%): proportion of fully developed fruits relative to total fruits. (**E**) Double kernel ratio (%): proportion of double-kernel fruits in the total number of fruits. (**F**) Kernel recovery rate (%): percentage of kernel weight relative to fruit weight after shelling. Data are presented as means ± standard error (SE) from three biological replicates. Different lowercase letters above the bars indicate significant differences among treatments at *p* < 0.05 according to Tukey’s honest significant difference (HSD) test.

Yield composition is a key factor for directly evaluating seed vigor and quality. The results showed that at a seed moisture content of 5%, the saturation rate, double kernel rate, kernel emergence rate, and yield per hectare were all low and decreased with decreasing storage temperature (Figure 3). Yield declined by 5.3%, 11.2%, and 22.2%, respectively. When the moisture content increased to 10%, yield decreased by 6.7%, 29.3%, and 36.5%, showing significant differences. Overall, the maximum yield occurred in the 0 °C/5% treatment, whereas the minimum yield was observed in the −10 °C/15% treatment, followed by the −40 °C/10% treatment.

### 2.5. Response Mechanism of Key Genes in Seeds Based on Transcriptome Analysis of Storage Temperature and Water Content

**Figure 4:**
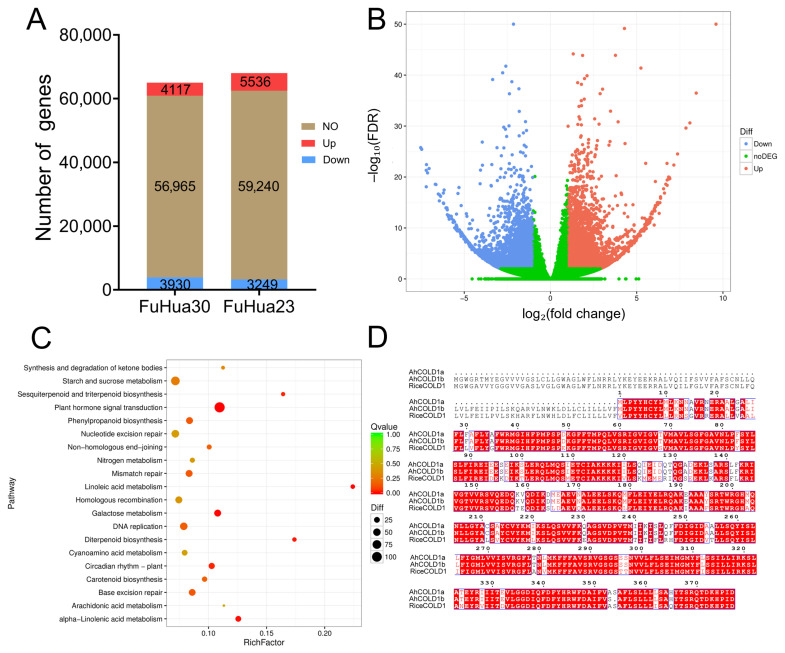
Differential gene expression analysis and functional enrichment results. (**A**) Bar chart showing the number of upregulated (red), downregulated (blue), and non-differentially expressed (brown) genes in fuhua30 and fuhua23. (**B**) Volcano plot illustrating the distribution of differentially expressed genes. The *x*-axis represents log_2_(fold change), and the *y*-axis represents −log_10_(FDR). Red points indicate upregulated genes, blue points indicate downregulated genes, and green points indicate non-significant genes. (**C**) Go pathway enrichment bubble plot. The *x*-axis represents the Rich Factor, and the *y*-axis lists the Go pathways. Bubble size corresponds to the number of genes, and color indicates the Q-value. (**D**) Sequence alignment of COLD1a, COLD1b, and Rice COLD1 proteins generated using sequences retrieved from UniProt, aligned with ClustalW, and visualized with ESPript.

To further investigate the molecular mechanism underlying frost resistance in peanut, frost treatment (–4 °C) and room temperature controls were applied to a frost-resistant germplasm (FH 30) and a non-frost-resistant germplasm (FH 23). After 12 h of treatment, samples were rapidly collected for transcriptome sequencing (Figure 4). Sequencing results revealed that 65,012 genes were detected in FH 30 and 68,025 genes in FH 23, accounting for 77.6% and 81.2% of the total peanut genes, respectively (Figure 4A). In FH 30, 4117 genes were upregulated and 3930 were downregulated, whereas in FH 23, 5536 genes were upregulated and 3249 were downregulated (Figure 4B). Differentially expressed genes in the frost-resistant FH 30 were further analyzed using volcano plots and Gene Ontology (GO) enrichment analysis. The GO enrichment results indicated significant enrichment in terms such as oxidation–reduction process, metabolic process reprogramming, ATP catabolic process, and regulation of transcription, DNA-templated (Figure 4C). These findings suggest that the frost resistance of FH 30 may be attributed to the activation of multiple stress-responsive pathways. In particular, the enrichment of oxidation–reduction and ATP catabolic processes may enhance the capacity to maintain redox balance and energy homeostasis under cold stress. Concurrently, transcriptional reprogramming mediated by cold stress-related transcription factors likely facilitates the rapid regulation of gene expression and metabolic status, thereby improving cold adaptation.

Further analysis and screening of upregulated differentially expressed genes in FH 30 identified 14 key candidates with high fold-change values that may be involved in the cold stress response (Table S1), including *FQR1-like2*, *COLD1*, and others. Notably, COLD1 has been reported to participate in cold stress responses in rice and Arabidopsis [22,23], suggesting that *AhCOLD1* may also play a role in the cold stress response of peanut.

### 2.6. Cloning of AhCOLD1a from Peanut and Functional Validation of Its Role in Cold Tolerance Through Overexpression in Arabidopsis thaliana

In 2015 [23], the cold tolerance gene *COLD1* was cloned in rice. This gene encodes a regulatory factor of the G protein signaling pathway and has been shown to enhance cold tolerance in rice. To further investigate the potential function of *COLD1* in peanut, two peanut COLD1 homologs, *AhCOLD1a* and *AhCOLD1b*, were identified through a combination of peanut genome analysis and molecular cloning. The *AhCOLD1a* gene is 10,321 bp in length, while *AhCOLD1b* is 9461 bp in length, encoding proteins of 377 and 468 amino acids, respectively. Protein structure analysis revealed that both AhCOLD1a and AhCOLD1b possess the conserved GPCR-type G domain (Figure 4D), suggesting that they may play a role in peanut cold tolerance.

A 35S::COLD1a-pSuper1300 expression vector was constructed via homologous recombination and introduced into Agrobacterium tumefaciens strain GV3101. Arabidopsis thaliana plants were transformed using the floral-dip method. Following surface sterilization, T0 seeds were sown on 1/2 MS medium supplemented with 30 mg/L hygromycin for selection. A total of 46 T1 transgenic lines were obtained based on rooting phenotype and resistance gene identification (Figure S1). After phenotypic segregation and screening, T3 homozygous seeds were obtained for four overexpression lines: 77-2, 78-1, 87-1, and 93-2. Under low-temperature treatment, *AhCOLD1a* overexpression lines exhibited a germination index (GI) of 6.69, a mean germination time (MGT) of 10.04 days, and a germination potential (GP) of 68.39%, representing significant improvements over the control. However, no significant difference in germination rate was observed between overexpression lines and controls. Under normal temperature conditions, no significant differences were detected for any germination parameters. These results indicate that *AhCOLD1a* overexpression can significantly shorten germination time, enhance germination vigor, and improve cold tolerance under chilling stress.

## 3. Discussion

### 3.1. Effects on Seed Vitality

As the first step in agricultural production, the quality and vitality of seeds directly affect the agricultural production [27]. Currently recognized environmental conditions that affect seed vitality and quality include water, temperature, microorganisms, and pests and diseases. Among them, temperature and water are the two main factors that directly affect seed quality and later use [6]. Previous studies have reported that once the moisture content of seeds exceeds the safe threshold, their lifespan will significantly decrease with the increase in moisture content. On the other hand, for every 5 °C increase in storage temperature, the lifespan of seeds also decreases severely [28]. However, the safe range of moisture content for seeds of different crops is not the same. In our experimental results, we found that at the same temperature, seed vitality significantly decreased with increasing water content (Figure 1). Among them, the germination rate of seeds under the −20 °C/15% and −40 °C/15% treatments was 0, which fully demonstrates the influence of water content on seed vitality. Further research on its principle may be due to the freezing of water between cells under low temperature conditions, causing mechanical damage to the cell membrane structure, thereby disrupting the normal physiological mechanism of cells and even causing seed death [29]. Currently, it is generally believed that the safe moisture content for peanuts should be between 5% and 10%, especially under high temperature and humidity conditions, which can easily lose vitality [30]. For example, Meng (1992) reported [31] that when the moisture content of peanut seeds exceeds 10%, they can still be frozen and spoiled even at −3 °C. So, the mechanical damage to cells caused by low temperatures and the cell swelling caused by high water content are the main reasons for the failure of seed vitality [32,33].

### 3.2. Effects on Seed Nutrient Composition and Content

Protein, as the most fundamental unit of life activity, plays an important role in seed germination and growth, including the exercise of enzyme functions and the provision of nitrogen elements. It is generally believed that there is a positive correlation between seed vitality and protein content, which has also been validated in various crops such as soybeans and rapeseed [34,35]. In this experiment, as the temperature and moisture content increased, the protein content gradually decreased (Table 1), which is consistent with reports in other studies. However, there are also reports to the contrary. Fan et al. (1993) [35] reported that as seed vitality is lost, protein content increases. As the main source of seed energy storage species, fat metabolism is directly related to seed germination and vitality. For example, excessive oxidation of unsaturated fatty acids can directly increase MDA content, ultimately destroying seed vitality. However, the impact of seed vitality and fat content is currently influenced by various factors such as crop types, and further research is needed to determine [36,37].

### 3.3. Effects on Antioxidant Enzyme Activity During Peanut Seedling Stage

During storage, seeds accumulate reactive oxygen species (ROS) and peroxides, which disrupt membranes and accelerate aging and even death [36,38]. SOD/POD/CAT, as the three most typical enzymes, have been extensively reported and validated [39]. In this experiment, the SOD enzyme activity reached its maximum at 0 °C/5% treatment (Figure 2A), but decreased with increasing water content, indicating that the increase in water content at the same temperature may accelerate the destruction of proteases in seedlings, while decreasing temperature directly affects enzyme activity. On the other hand, the content first increases and then decreases with seedlings grow, which may be related to plant growth activity [40]. The activity trend of POD followed a similar trend to that of SOD (Figure 2C). At the same temperature, with the increase in water content, its enzyme activity actually decreases, which may be directly related to the changes in seed quality and vitality [41]. It is interesting that MDA, as a key indicator of the degree of cell membrane damage, also follows the trend of enzyme activity changes, and its minimum value was found under the 0 °C/5% treatment (Figure 2D), indicating that the degree of cell membrane damage was the smallest under this treatment. Overall, with the increase in water content and the decrease in storage temperature, the MDA content showed an upward trend. However, in this experiment, the maximum MDA value was found in the −10 °C/15% treatment, which may indicate that the changes in MDA content are influenced by multiple factors, including sampling time, etc., and its changes may not increase linearly, but be jointly driven by multiple factors [42,43].

### 3.4. Impact on Peanut Yield Composition

Yield is one of the most critical indicators for evaluating seed quality. In this experiment, the yield varied with changes in seed storage temperature and moisture content (Figure 3). So, seed treatment with good quality and high vitality will achieve the maximum yield [9]. In this experiment, the maximum yield was obtained at 0 °C/5% treatment, which was positively correlated with the increase in nutrient content, enzyme activity, and cell membrane integrity (Figure 1, Figure 2, Figure 3 and Figure 4). Further indicating that this treatment has the best effect on maintaining seed vitality. Ebone et al. (2020) [44] also found that under the same cultivation techniques, the higher the vitality of soybean seeds, the greater the yield obtained. This is also consistent with the results of our current experiment. On the other hand, the yield was the lowest under the −10 °C/15% treatment, mainly due to its low emergence rate (Figure 1). Although the number of results and kernel emergence rate are not significantly different, the low emergence rate seriously affects the yield. This further reflects the joint influence of multiple factors on yield in actual field production, including not only seed quality, but also water and fertilizer management, cultivation management, and so on. Therefore, it is necessary to comprehensively evaluate various factors that affect yield [44].

### 3.5. Differential Expression Genes in Response to Cold Stress and the Function of COLD1

The transcriptome sequencing results showed that 4117 genes were upregulated and 3930 genes were downregulated in FH30 and 5536 upregulated genes and 3249 downregulated genes in FH23, GO enrichment analysis showed that among the differentially expressed genes of FH30, functions such as “oxidation-reduction process”, “metabolic process reprogramming”, “ATP catabolic process”, and “regulation of transcription (DNA templated)” were significantly enriched, suggesting that its frost resistance may be closely related to the activation of multiple stress response pathways (Figure 4). Gu. et al., (2023) [45] also found that differentially expressed genes in wheat with different cold resistance were enriched in oxidation-reduction process term. These pathways may help plants maintain redox homeostasis and regulate energy metabolism under low temperature stress, and achieve rapid regulation of gene expression and metabolic status through the activation of cold stress-related transcription factors, thereby enhancing their ability to adapt to low temperatures [45,46]. On the other hand, In this study, transcriptome analysis of peanut under cold stress revealed an upregulation of the *COLD1* gene. This expression pattern closely mirrors the low-temperature responses of *COLD1* in rice and Arabidopsis [23,25], and the consistency of these results suggests that the gene may play a conserved role in the cold stress response of peanut. Cloning of *AhCOLD1a* and *AhCOLD1b* showed that both proteins retain a conserved GPCR-type G domain, further supporting the functional conservation of the *COLD1* family during evolution. This domain, as a critical component of the heterotrimeric G protein signaling pathway, is likely to mediate low-temperature signal perception and transmission in peanut through a mechanism similar to the interaction between rice COLD1 and G protein [24].

### 3.6. Possible Downstream Regulatory Mechanisms and Application Value of COLD1

Further combined with GO enrichment analysis, it is speculated that the cold resistance mechanism of *AhCOLD1a* may involve synergistic regulation of multiple pathways. The enrichment of redox processes suggests that it may maintain the steady-state balance of reactive oxygen species (ROS) at low temperatures by activating antioxidant enzyme systems such as SOD and POD, or regulating NADPH oxidase activity. This may be similar to the mechanism by which *COLD1* in rice activates downstream antioxidant responses through calcium ion channels. The enrichment of ATP decomposition related genes points to the reprogramming of energy metabolism: at low temperatures, cell energy supply is limited, and *AhCOLD1a* may prioritize cold resistance related processes such as cell membrane repair and synthesis of osmoregulatory substances by regulating energy allocation, thereby maintaining the stability of cell structure and function. It is worth exploring that the G protein signaling pathway itself has the ability to integrate environmental signals with intracellular metabolic states. Therefore, *AhCOLD1a* may integrate cold stress signals with redox homeostasis and energy metabolism demands through a GPCR type G domain mediated signaling network, forming a cascade regulation of “perception conduction response”. This hypothesis needs further verification through protein interaction experiments and metabolomics analysis. From an application perspective, the discovery of *AhCOLD1a* provides a new molecular target for peanut cold resistant breeding. Compared to the phenotype selection relied on in traditional breeding, molecular assisted selection using *AhCOLD1a* as a marker can significantly improve the efficiency of cold resistant germplasm screening; By optimizing its expression pattern or protein activity through gene editing technology, it is expected to improve the low-temperature adaptability of peanuts in a targeted manner.

From the perspective of practical agricultural applications, storing peanut seeds at 0 degrees Celsius has the best effect on seed vitality and subsequent yield. Therefore, we recommend that local farmers store seeds under this condition. Although there are certain shortcomings in this experiment (short storage period, constant rather than fluctuating conditions, limited number of cultivators, heterogeneous validation in Arabidopsis), the results of this experiment can still fully reflect some underlying mechanisms. For farmers without relative storage conditions, they should at least avoid relatively high moisture content and low-temperature freezing damage, at least not 15% moisture content. On the other hand, the function of the ahcold1 gene has been validated to some extent in Arabidopsis, but further validation is needed in peanuts in the future, which may provide a clearer understanding of the gene’s function.

## 4. Materials and Methods

### 4.1. Plant Materials

The peanut varieties Fu Hua 30 (FH30) and Fu Hua 23 (FH23) were used in this study. Seed germination and seedling stage experiments were conducted separately, with FH30 being a cold-resistant variety and FH23 a cold-sensitive variety. All materials were independently cultivated and preserved by our research institute.

### 4.2. Experimental Sites

The experiment was conducted in the experimental field of the scientific research demonstration base in Fuxin City (Xihe District, Fuxin City, E 121°01′–122°25′; N 41°44′–42°34′). The experimental area was located in a transitional zone from the northern temperate sub-humid climate to the semi-arid continental monsoon climate, belonging to a semi-arid region. The soil was classified as sandy soil, with a pH of 5.36, a bulk density of 1.46 g·cm^−3^, an organic matter content of 15.8 g·kg^−1^, a total nitrogen content of 0.39 g·kg^−1^, an available phosphorus content of 36.6 mg·kg^−1^, and an available potassium content of 17.2 mg·kg^−1^.

### 4.3. Treatments and Experimental Design

After air drying and adjusting the water content of the harvested peanut varieties, the seeds were divided into three groups (5%, 10%, and 15% moisture content) and stored at four temperatures (0 °C, −10 °C, −20 °C, and −40 °C) for 2 months before being kept at room temperature for future use. In total, four storage temperatures × three moisture levels resulted in 12 treatments. Randomized complete block design with three replications was used, with each plot consisting of 4 ridges (6.3 m long, 17.5 cm hole spacing, 45 cm row spacing, 2 seeds per hole). Seed vitality was tested before sowing, physiological indicators and transcriptome sequencing were conducted at the seedling stage, transgenic Arabidopsis was constructed to validate gene function, and peanut yield was measured to evaluate the effects of different treatments.

### 4.4. Indicator Determination and Data Collection

Seed quality and vitality indicators were measured before sowing. After sowing and emergence, fresh leaves at the same leaf position were collected from different treatments according to the growth cycle for enzyme activity indicator testing, with three replicates per test.

The moisture content of seeds was determined by high-temperature drying method. The fat, protein, oleic acid, and linoleic acid in the seeds were measured using VEC-TOR22/N-type Fourier transform near-infrared spectrometer. The germination rate and germination potential of the seeds were determined using the standard germination rate measurement method of the International Seed Inspection Regulations [47]. The activities of peroxidase (POD) and catalase (CAT) were measured using the guaiacol method and Chance method, respectively. The activities of superoxide dismutase (SOD) and malondialdehyde (MDA) content were determined using the detection kit according to the manufacturer’s instructions, respectively [32].

Microsoft Excel 2003 was used to organize and plot the data. All data in the graph were mean ± standard deviation (SE), and SPSS 16.0 was used for multivariate statistical analysis. One way ANOVA (Duncan test) was used for significance difference analysis.

Transcriptome sequencing and data analysis: Pods of frost-resistant (FH30) and frost-sensitive (FH23) peanut varieties with uniform maturity were selected and subjected to frost treatment (−4 °C) or room temperature control (25 °C) for 12 h. Embryonic roots and germ tissues were collected, rapidly frozen in liquid nitrogen, and used for RNA extraction. Three biological replicates were set for each treatment, totaling 12 samples. RNA sequencing was performed on the Illumina NovaSeq 6000 platform. Quality control of raw reads was conducted using FastQC, and clean reads were aligned to the peanut reference genome (Tifrunner.gnm2.J5K5, https://data.legumeinfo.org/Arachis/hypogaea/genomes/Tifrunner.gnm2.J5K5/, (accessed on 18 August 2024). Gene expression levels were quantified as FPKM, and differentially expressed genes (DEGs) were identified using DESeq2 (v1.30.1) with |log2FoldChange| ≥ 1 and adjusted *p*-value < 0.05. DEGs were further subjected to GO and KEGG enrichment analysis. All sequencing and data analysis were conducted by Shenzhen Hengchuang Biotechnology Co., Ltd. (Shenzhen, China).

To investigate the function of the *AhCOLD1a* gene in plants, a 35S::COLD1a-pSuper1300 plant expression vector was constructed using homologous recombination. The recombinant plasmid was introduced into Agrobacterium tumefaciens strain GV3101. Wild-type Arabidopsis thaliana (Col-0) plants were transformed via Agrobacterium-mediated floral dip, following a streamlined protocol described by Davis et al. (2009) [48]. Briefly, flowering Arabidopsis inflorescences were dipped in the bacterial suspension containing the target vector. Seeds were harvested after maturation, surface-sterilized, and sown on 1/2 MS medium containing hygromycin for selection. Positive T_1_ transformants were confirmed by PCR analysis.

### 4.5. Statistical Analysis

Microsoft Excel 2003 was used to organize and plot the data. All data in the graph were mean ± standard deviation (SE), and SPSS 16.0 was used for multivariate statistical analysis. One way ANOVA (Duncan test) was used for significance difference analysis.

## 5. Conclusions

This experiment investigated the effects of seed storage temperatures and moisture treatments on peanut yield. The results indicate that high moisture content can seriously reduce seed vitality or cause seed death, ultimately resulting in a significant decrease in actual yield. In this experiment, the storage temperature of 0 degrees Celsius achieved the highest seed quality and yield in summer. Therefore, it is recommended to control the seed storage conditions as much as possible under this condition, at least avoiding excessive moisture content (15%). However, this experiment also has certain limitations, including short processing time and limited variety, so further verification needs to be carried out in conjunction with the main cultivated varieties in the local area. In addition, we performed transcriptome sequencing and identified a resistance gene AhCOLD1. The results of transgenic Arabidopsis indicate that overexpression of this gene can enhance plant resistance to cold stress. However, due to heterologous expression, this experiment still has certain limitations. In the future, it is necessary to further expand the testing of peanut varieties and conduct verification of genetically modified peanut plants, in order to provide more technical support for future peanut production.

## Figures and Tables

**Table 1:** Changes in protein, fat, oleic acid, and linoleic acid contents in peanut seeds under different storage temperatures and moisture levels.

Treatment	Protein	Fat	Oleic Acid Content	Linoleic Acid Percentage
	(%)	(%)	(%)	(%)
0 °C/5%	25.37 ± 1.26	50.13 ± 2.03	42.95 ± 0.15	39.77 ± 4.06
0 °C/10%	25.32 ± 1.07	49.92 ± 1.60	41.48 ± 1.36	39.5 ± 4.13
0 °C/15%	25.1 ± 0.69	49.85 ± 3.62	38.91 ± 0.15	38.15 ± 1.28
−10 °C/5%	25.67 ± 2.01	49.91 ± 4.15	42.11 ± 0.26	39.73 ± 2.34
−10 °C/10%	25.14 ± 3.04	49.88 ± 0.29	40.33 ± 1.36	39.16 ± 1.29
−10 °C/15%	25.04 ± 4.26	48.82 ± 2.36	32.49 ± 2.31	35.7 ± 0.38
−20 °C/5%	24.69 ± 0.98	49.99 ± 4.12	41.47 ± 2.16	39.5 ± 1.66
−20 °C/10%	25.1 ± 2.34	49.93 ± 2.36	39.46 ± 2.67	39.07 ± 2.34
−20 °C/15%	24.92 ± 1.26	47.8 ± 5.40	28.17 ± 5.15	33.14 ± 2.06
−40 °C/5%	25.14 ± 5.10	50.64 ± 1.92	40.09 ± 2.16	38.92 ± 0.34
−40 °C/10%	25.47 ± 3.08	49.73 ± 6.01	37.18 ± 3.19	38.73 ± 3.01
−40 °C/15%	24.36 ± 6.05	46.52 ± 5.21	26.21 ± 4.16	32.36 ± 6.41

Values are expressed as mean ± standard deviation (n = 3).

## Data Availability

The original contributions presented in the study are included in the article material. Further inquiries can be directed to the corresponding author.

## Supplementary Materials

The following supporting information can be downloaded at: https://www.mdpi.com/article/10.3390/plants14192944/s1. Table S1. Changes in protein, fat, oleic acid, and linoleic acid contents in peanut seeds under different storage temperatures; Figure S1. Hygromycin resistance screening, molecular identification, and growth performance of transgenic plants. (A) Rooting assay of T_0_ generation seedlings under hygromycin selection. Red boxes indicate seedlings with hygromycin resistance and normal root development. (B) PCR-based identification of hygromycin-resistant T_1_ generation plants. The expected product size is indicated by the arrow. (C) Growth status of transgenic plants after transplantation under normal temperature conditions. (D) Mature transgenic plants at the reproductive growth stage; Figure S2. Principal component correlation analysis. Conducting correlation analysis on key indicators, group A means 0 °C, group B means −10 °C, group C means −20 °C, and group D means −40 °C. The arrow represents the main direction after dimensionality reduction analysis, and the angle represents the degree of correlation.
