# Supplementary material for: The Impact of Storage Conditions on Peanut Seed Quality, Growth, and Yield
Source: Plants (Basel). 2025 Sep 23;14(19):2944. doi: 10.3390/plants14192944 (PMC12526394; doi:10.3390/plants14192944)
Supplement: Supplementary file 1 [file plants-14-02944-s001.zip › plants-3842725-supplementary.pdf]

Table S1 Changes in protein, fat, oleic acid, and linoleic acid contents in peanut seeds under different storage temperatures

GeneID	log2Fold	Annotation
	11.11	Cyclin-dependent kinase F-4 OS=Oryza sativa subsp. japonica (Rice) PE=2 SV=1
LARP1B	8.21	La-related protein 1B GN=LARP1B OS=Arabidopsis thaliana (Mouse-ear cress) PE=2 SV=1
F20M17.9	7.55	Poly(ADP-ribose) glycohydrolase 1 GN=F20M17.9 OS=Arabidopsis thaliana (Mouse-ear cress) PE=1 SV=2
B1114D08.16-1	7.54	DEAD-box ATP-dependent RNA helicase 5 GN=B1114D08.16-1 OS=Oryza sativa subsp. japonica (Rice) PE=2 SV=1
	7.48	Zinc finger CCCH domain-containing protein 44 GN=At3g51120 OS=Arabidopsis thaliana (Mouse-ear cress) PE=2 SV=3
F4D11.140	7.46	Serine/threonine-protein kinase AFC3 GN=F4D11.140 OS=Arabidopsis thaliana (Mouse-ear cress) PE=2 SV=2
PRR73	7.23	Two-component response regulator-like PRR73 GN=PRR73 OS=Oryza sativa subsp. japonica (Rice) PE=2 SV=1
FQR1-like 2	7.21	Probable NAD(P)H dehydrogenase (quinone) FQR1-like 2 {ECO:0000305} OS=Arabidopsis thaliana PE=1 SV=1
	7.14	Protein transport protein Sec61 subunit alpha OS=Pyrenomonas salina PE=2 SV=1
	7.11	Glutamyl-tRNA(Gln) amidotransferase subunit A, chloroplastic/mitochondrial OS=Oryza sativa subsp. japonica (Rice) PE=3 SV=3
SC35	7.10	Serine/arginine-rich splicing factor SC35 GN=SC35 OS=Arabidopsis thaliana (Mouse-ear cress) PE=1 SV=1
COLD1a	7.10	COLD1a-like protein OS=Arachis hypogaea PE=2 SV=1
	7.09	Protein transport protein SEC16A homolog {ECO:0000305} OS=Arabidopsis thaliana (Mouse-ear cress) PE=1 SV=1
	7.08	Zeaxanthin epoxidase, chloroplastic (Precursor) OS=Prunus armeniaca (Apricot) PE=2 SV=1

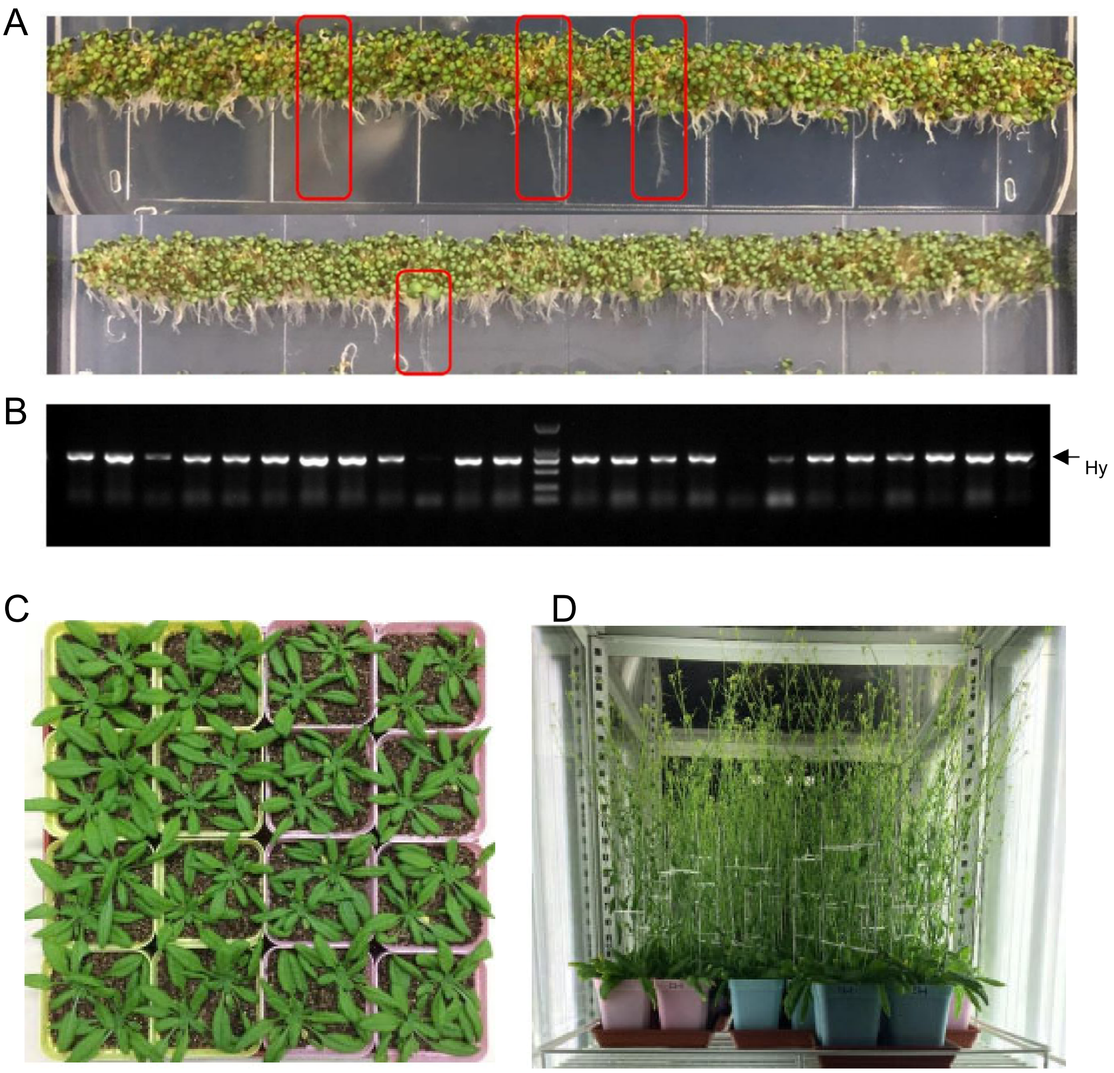

Figure S1. Hygromycin resistance screening, molecular identification, and growth performance of transgenic plants.(A) Rooting assay of T_0 generation seedlings under hygromycin selection. Red boxes indicate seedlings with hygromycin resistance and normal root development.(B) PCR-based identification of hygromycin-resistant T_1 generation plants. The expected product size is indicated by the arrow.(C) Growth status of transgenic plants after transplantation under normal temperature conditions.(D) Mature transgenic plants at the reproductive growth stage.

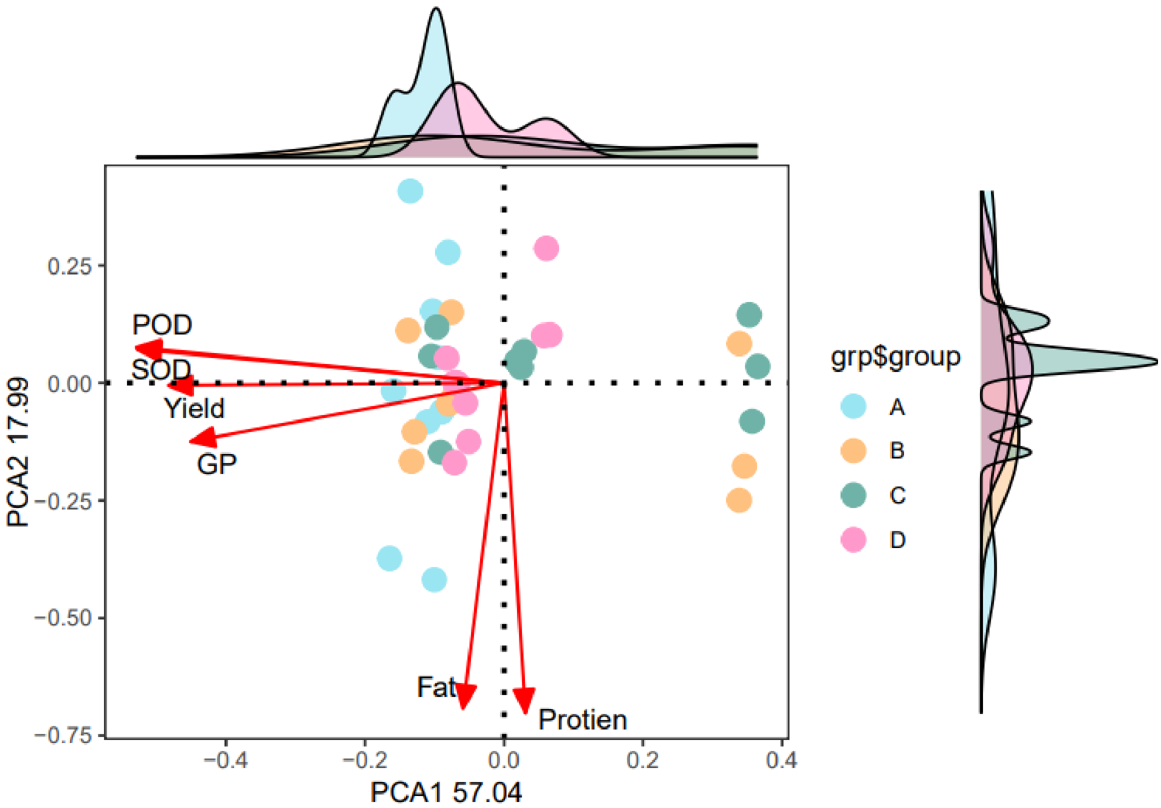

Figure S2**.** Principal component correlation analysis. Conducting correlation analysis on key indicators, group A means 0 ℃, group B means -10 ℃, group C means -20 ℃, and group D means -40 ℃. The arrow represents the main direction after dimensionality reduction analysis, and the angle represents the degree of correlation.
